# A Guanidine-Based Superbase as Efficient Chemiluminescence Booster

**DOI:** 10.1038/s41598-019-51105-z

**Published:** 2019-10-10

**Authors:** Christina M. Geiselhart, Christian W. Schmitt, Philipp Jöckle, Hatice Mutlu, Christopher Barner-Kowollik

**Affiliations:** 1 Soft Matter Synthesis Laboratory, Institut für Biologische Grenzflächen, Karlsruhe Institute of Technology (KIT), Hermann-von-Helmholtz-Platz 1, 76344 Eggenstein-Leopoldshafen, Germany; 2 Macromolecular Architectures, Institut für Technische Chemie und Polymerchemie, Karlsruhe Institute of Technology (KIT), Engesserstraße 18, 76128 Karlsruhe, Germany; 3 School of Chemistry, Physics and Mechanical Engineering and Institute of the Future Environments, Queensland University of Technology (QUT), 2 George Street, QLD, 4000 Brisbane, Australia; 4 Molecular Physical Chemistry, Institute of Physical Chemistry, Karlsruhe Institute of Technology (KIT), Fritz-Haber-Weg 2, 76131 Karlsruhe, Germany

**Keywords:** Analytical chemistry, Organic chemistry

## Abstract

We introduce the guanidine-based superbase 1,5,7-triaza-bicyclo-[4.4.0]dec-5-ene (TBD) as efficient enabler for chemiluminescence (CL) based on luminol in a simple, ready-to-use two component system. The strong CL is generated by the superbase’s properties as peroxidase mimetic and bifunctional coreactant. The herein established concept allows for CL enabling molecules (superbases) to be readily implemented in larger molecular structures, including in polymers.

## Introduction

**Figure 1:**
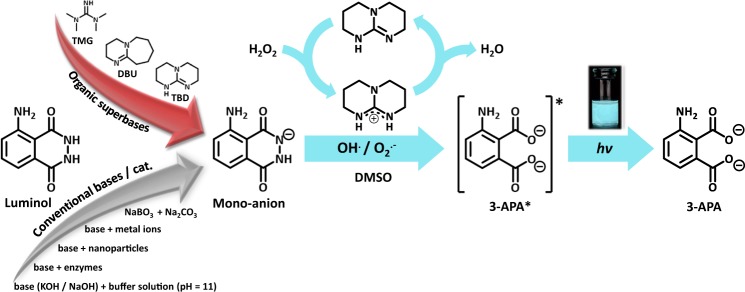
Chemiluminescence reaction of luminol in the presence of a base (and catalyst) triggered by H_2_O_2_, leading to the formation of 3-aminophthalic acid (3-APA). Next to previously used bases and catalysts, the structures of the organic superbases TBD, TMG and DBU are displayed.

Superbases have proven to be outstanding catalysts in organic chemistry by virtue of their simple molecular modification, possible recyclability, and lower toxicity^1,2^. Thus, they have been implemented in a wide range of organic reactions, (e.g. cycloadditions^3^, Michael addition^4,5^, recyclization reactions^6^, Henry reactions^1^, Wittig and Horner-Wadsworth-Emmons reactions^7^) as well as in ring-opening polymerizations^8,9^. Specifically, the cyclic nitrogen-containing organosuperbase 1,5,7-triaza-bicyclo-[4.4.0]dec-5-ene (TBD, p*K*_a_ value 26.0 in acetonitrile^10^) displays a rich and unique chemistry conferred by its specific structure and binding properties (hydrogen bond donor and acceptor activity)^10–13^. In addition, the incorporated guanidine functionality, which is present in several natural compounds (e.g. agmatine^14^ or arginine^10^), makes it interesting for peptide mimicking due to the ability to bind carboxylates and phosphates^15,16^. Due to the free guanidine moiety in the aforementioned aliphatic compounds, electron delocalization takes place and the guanidine functionality is converted to the respective urea functionality under oxidative conditions (refer to Supplementary Fig. S1). By taking a substituted guanidine moiety embedded into a bicyclic scaffold, i.e. TBD, the steric hindrance prevents the delocalization as well as the oxidation to the urea compound. Thus, TBD ideally lends itself as catalyst under oxidative conditions. Inspired by both the basic and catalytic properties of the commercially available TBD, we aimed to establish a new, simple two-component chemiluminescence (CL) system. CL reactions have been exploited in variable fields^17–21^, exploiting its high sensitivity, rapidity, wide dynamic range, controllable emission rate, relatively simple handling, and low instrumentation costs^22^. Among the plethora of CL luminophores, luminol has perhaps attracted the most scientific interest owing to its low cost, favourable properties, compatibility with a large number of analytes and a broad range of applications^23–27^. However, in contrast to bioluminescence systems, the CL emission generated during the oxidation of luminol is of relatively low intensity due to its low quantum yield (≈0.01 in aqueous media and ≈0.09 in DMSO)^28,29^. Therefore, the enhancement of the inherent CL emission is a key factor if luminol is to be exploited in e.g. macromolecular self-reporting systems. In fact, over the past few decades the toolbox of catalyst systems employed in the luminol CL reaction has been expanded from classical systems including metal ions^30^ and enzymes^31^ to more sophisticated variants such as nanomaterials^32^. However, each of the aforementioned systems suffers from specific disadvantages. For instance, metal catalysts may be expensive, toxic and sensitive to air or moisture. Likewise, sensors based on enzymes are fraught with issues that include low reproducibility and limited stability and shelf-life^33^. On the other hand, the catalytic activity of nanoparticles during CL significantly depends on particle size and distribution^32^. Furthermore, harsh conditions with a high excess of base, ranging from 20 to 1000 equivalents^34^, is required. As a consequence, there exists a strong continuing interest in the development of new catalysts and environments for luminol CL, which can overcome the aforementioned critical limitations and widen its applications. Given TBD’s inherent nucleophilicity and hydrogen-bonding capability, we hypothesized a strong effect on the luminol CL reaction, while concomitantly allowing to execute the reaction in organic media (specifically in DMSO, which possesses a relatively benign toxicity profile), since the use of non‐aqueous organic media is of increasing importance in several biotechnological applications to achieve process enhancement^35–37^. Critically, the yield and the colour of the luminol CL emission are dependent on the nature of the solvent^38–40^. CL in alkaline aqueous solution is violet-blue (λ_em_ = 425 nm), while in neutral dimethylsulfoxide (DMSO)^41^ it is centered at 405 nm. Alternatively, when an inorganic base^9^ (i.e. KOH) is added to the luminol-DMSO systems, CL with an emission maximum of 485 nm with a blue-green colour is observed. Recently, Watanbe *et al*. reported the successful application of organic superbases in the CL reaction of dioxetanes^42^, encouraging us to expand the scope of the luminol CL reaction enabled by organosuperbases. A recent survey of the literature suggests that the activity of any organic superbase in the luminol CL system (either in aqueous or organic media) has not been exploited. In an effort to demonstrate the validity of our luminol-superbase-concept, along TBD we investigated two other well-known superbase derivatives, i.e. 1,1,3,3-tetramethylguanidine (TMG, p*K*_a_ value 23.3 in acetonitrile^10^) and 1,8-diazabizyclo[5.4.0]undec-7-ene (DBU, p*K*_a_ value 23.9 in acetonitrile^10^) (refer to Fig. 1). Our herein presented study of the superbase/luminol CL systems is underpinned by detailed analytical characterization employing chemiluminescence, EPR, 1D/ 2D NMR and UV/Vis spectroscopy as well as GC-MS spectrometry. For comparison with already established luminol-CL-systems, measurements of luminol with KOH and KOH/CuSO_4_, an inorganic base and catalyst already known in the context of luminol-CL, were conducted^34,43^. All experiments were conducted in DMSO.

## Results and Discussion

**Figure 2:**
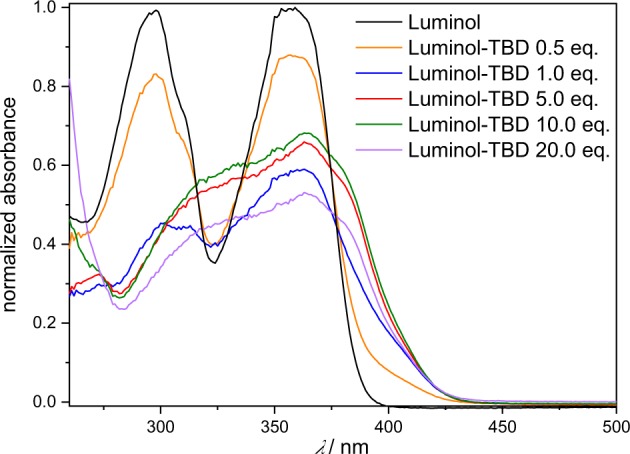
UV/Vis spectra of luminol (*c* = 7.5 × 10^−5^ mol L^−1^) in DMSO with different concentrations of TBD (*c*(0.5 eq.) = 3.75 × 10^−5^ mol L^−1^, *c*(1.0 eq.) = 7.5 × 10^−5^ mol L^−1^, *c*(5.0 eq.) = 37.5 × 10^−5^ mol L^−1^, *c*(10.0 eq.) = 75.0 × 10^–5^ mol L^−1^, *c*(20.0 eq.) = 150.0 × 10^−5^ mol L^−1^).

**Figure 3:**
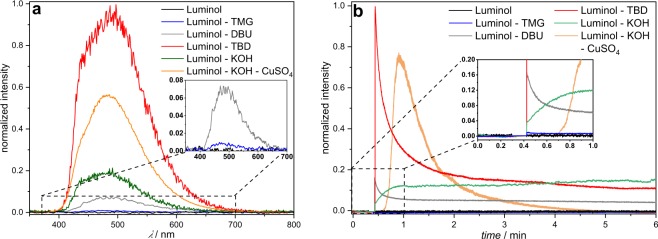
(**a**) CL emission of luminol-base-systems in DMSO (*c*(luminol) = 7.5 × 10^−2^ mol L^−1^, *c*(base) = 37.5 × 10^−2^ mol L^−1^, *c*(CuSO_4_) = 4.5 × 10^−2^ mol L^−1^) at ambient temperature, triggered by 0.1 mL H_2_O_2_ (1 mol L^−1^). (**b**) Time dependent CL emission of luminol-base-systems in DMSO (*c*(luminol) = 7.5 × 10^−2^ mol L^−1^, *c*(base) = 37.5 × 10^−2^ mol L^−1^, *c*(CuSO_4_) = 4.5 × 10^−2^ mol L^−1^, emission wavelength = 480 nm) at ambient temperature, triggered by 0.1 mL H_2_O_2_ (1 mol L^−1^).

**Figure 4:**
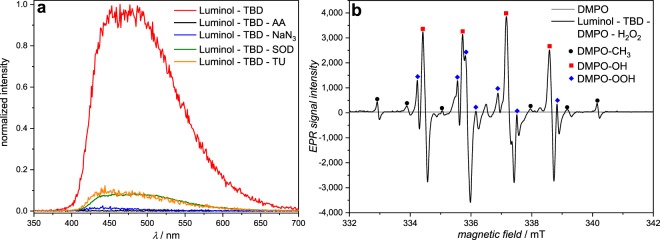
(**a**) CL emission of the luminol-TBD-system (*c*(luminol) = 7.5 × 10^−2^ mol L^−1^, *c*(TBD) = 37.5 × 10^−2^ mol L^−1^) in DMSO in the presence of radical scavengers (*c* = 2.5 × 10^−2^ mol L^−1^) at ambient temperature, triggered by H_2_O_2_. (**b**) EPR spectra of DMPO (grey line) and the luminol-TBD-system with DMPO after the addition of H_2_O_2_ (black line). The signals can be assigned to the different DMPO-radical-products: DMPO-CH_3_ (black dot), DMPO-OH (red square) and DMPO-OOH (blue rhomb).

**Figure 5:**
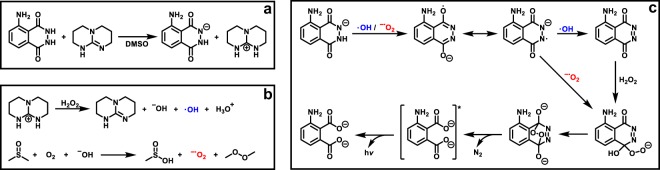
(**a**) (De-)Protonation of luminol and TBD in DMSO. (**b**) Generation of ROS by reaction of TBDH^+^ with H_2_O_2_, regeneration of TBD. DMSO reacts with O_2_ in the solution and the hydroxyl anions, formed during the reaction of TBDH^+^ with H_2_O_2_. (**c**) Oxidation of the luminol mono-anion by the formed ROS, leading to the emission of visible light.

**Figure 6:**
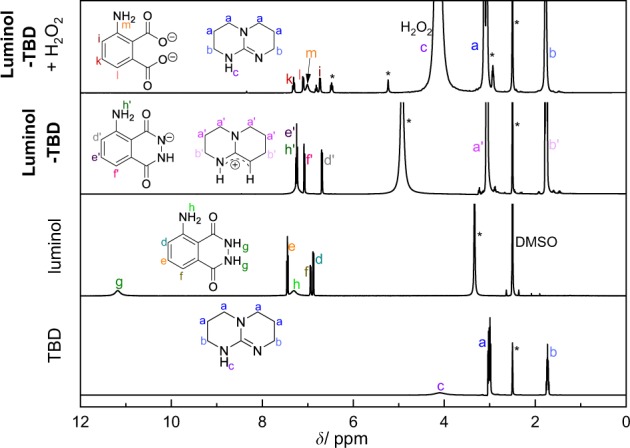
^1^H NMR (400 MHz) spectra of luminol, TBD, luminol-TBD and luminol-TBD + H_2_O_2_ in DMSO-d6.

First, the effect of the superbase-concentration on the luminol-CL-reaction was investigated by recording UV-Vis and CL emission spectra with different equivalents of TBD. While the UV/Vis spectra depicted in Fig. 2 indicate a significant change in the absorption between 1.0 eq. and 5.0 eq., the loading of TBD with 5.0, 10.0 or 20.0 eq. did not play a role. Addition of TBD > 1.0 eq. (red/green/purple line in Fig. 2) resulted in a notable change from a colorless to a yellow solution, which is associated with a decrease in the absorption band at 295 nm and 357 nm and simultaneous appearance of new bands at 329 nm and 370 nm. These observations are consistent with the formation of the luminol-mono-anion species^44^, suggesting that no mono-anions are formed in the presence of TBD < 5.0 eq. This behaviour is in agreement with the results from the CL emission measurements (see Supplementary Fig. S2). No CL emission for 0.5 and 1.0 eq. of TBD was recorded, while for ≥ 5.0 eq. of TBD a strong CL emission was obtained. To trigger the CL of the reaction, 0.1 mL of H_2_O_2_ (1 mol L^−1^) were added to the particular reaction mixture. Since there is no significant difference in the CL emission intensity for ≥ 5.0 eq. of TBD, the loading of the (super)base was maintained at 5.0 eq. To obtain an impression of the influence of the different (super)bases for the luminol-CL-reaction, the UV-Vis spectra of luminol with 5.0 eq of TMG, DBU and KOH were recorded. The spectra are shown in Supplementary Fig. S3. In the presence of one of the (super)bases, the absorption bands of luminol (black line) at 295 nm and 357 nm decrease, while simultaneously new bands at 329 nm and 370 nm appear, as described before for the UV-Vis spectra of different TBD-eq. in Fig. 2. Depending on the (super)base, the separate bands vanish, becoming more indistinct. The similarity in the absorbance between TBD (red line) and KOH (green line) in contrast to TMG (blue line) and DBU (grey line) suggest the formation of the luminol mono-anion in a more efficient way than in the presence of TMG and DBU. In order to investigate the actual CL of the luminol-(super)base systems, the CL emission intensities were measured. The spectra are depicted in Fig. 3a. Clearly, there is no CL when H_2_O_2_ is added to a solution of luminol without the addition of a coreactant (black line in Fig. 3a). In the presence of TBD however (red line in Fig. 3a), CL can be observed with an emission maximum at 480 nm, being five times higher than the CL emission of the luminol-KOH system (green line in Fig. 3a) and about two times higher than with an additional catalyst, namely CuSO_4_ (orange line in Fig. 3a). Additionally, the CL intensity of the luminol-TBD-system is 15x higher than the luminol-DBU-system (grey line in Fig. 3a) and 70x higher than the one in the luminol-TMG-system (blue line in Fig. 3a), respectively. To further investigate the CL characteristics of the luminol- superbase systems, the duration of the CL after the addition of H_2_O_2_ was measured (refer to Fig. 3b). The first 20 s were recorded without H_2_O_2_, then 0.1 mL H_2_O_2_ (1 mol L^−1^) were added. Again, no CL is observed when only luminol is present in the mixture (black line in Fig. 3b). In the presence of one of the organic superbases, the CL intensity increases immediately after the addition of H_2_O_2_ (refer to the blue, grey and red lines in Fig. 3b). Similar to the CL emission in Fig. 3a, TBD displays the highest initial CL intensity, namely 149 times. higher than the initial intensity of TMG and 6 times higher than the one of DBU. In the first minute after the H_2_O_2_ addition, the intensity decreases rapidly, while it decreases more slowly afterwards. Thus, the measurements were ceased after 6 min, since the immediate increase of the intensity after the addition of H_2_O_2_ evidences the suitability of the superbases for the CL of luminol and there is no significant change in the intensity anymore after 6 minutes. On the other hand, the intensity in the presence of KOH (green line in Fig. 3b) reaches its maximum one minute after the addition of H_2_O_2_ without a decrease within the next 5 min. However, the intensity of the luminol-KOH system is 37 times lower than the intensity of the luminol-TBD system immediately after the addition of H_2_O_2_ and approx. 8 lower at the maximum intensity. Interestingly, the CuSO_4_-catalyzed luminol-KOH system (orange line in Fig. 3b) shows a similar CL emission behaviour as the superbase-supported systems, yet with a delay of approx. 0.3 min after the addition of H_2_O_2_ and a 1.27-lower CL emission than the luminol-TBD-system, finishing after 5 min. Thus, the newly introduced luminol-TBD system outperforms the luminol-KOH (-CuSO_4_) system as well as the luminol-TMG and luminol-DBU systems. The obtained results are perfectly in line with recently published results from Watanabe *et al*., who investigated the CL reaction of dioxetanes induced by organic superbases^42^. Similar to our result, TBD proved to be more efficient than other organic superbases, e.g. TMG or DBU. Although a different luminophore (dioxetane) has been used in their work, comparable results for the efficiency of organic superbases (TBD > DBU > TMG) are obtained, however only 5 eq. of superbase are required for the luminol-CL instead of 200 eq. for the dioxetane-CL^42^. In contrast to the charge-transfer-induced decomposition mechanism of the dioxetane-CL-reaction^42^, the CL-reaction of luminol is triggered by reactive oxygen species (ROS)^29,45,46^. Typical ROS in the luminol-CL-reaction are the hydroxyl radical ^•^OH, the superoxide anion radical O_2_^•−^ or singlet oxygen ^1^O_2_^29,47,48^. To identify the involved ROS in the new luminol-TBD-system, spin-trap-experiments with the radical scavengers L-ascorbic acid (AA), sodium azide (NaN_3_), superoxide dismutase (SOD), thiourea (TU), 2,2,6,6- tetramethyl-4-piperidone (TMPD) and 5,5-dimethyl-1-pyrroline N-oxide (DMPO) were carried out^48^. As depicted in Fig. 4a, the CL emission decreases drastically in the presence of a radical scavenger (black/blue/green/orange line in Fig. 4a), proving the generation of ROS in the CL-reaction of the luminol-TBD-system. While SOD is known to be a specific radical scavenger for O_2_^•−^, NaN_3_ and TU are less specific, reacting with ^•^OH, O_2_^•−^ and ^1^O_2_^48–51^. Therefore, electron-paramagnetic resonance (EPR) spectroscopy was used to distinguish between the possible involved ROS. Due to the short lifetime of the ROS, DMPO and TMPD are used as spin-trap-compounds. Both compounds are not EPR active, unless they react with ROS to form the EPR-active nitroxide radicals, which possess a longer lifetime than ^•^OH, O_2_^•−^ or ^1^O_2_^48^. TMPD is a well-known spin-trap molecule for ^1^O_2_, while DMPO has been used for detection of ^•^OH and O_2_^•−^. The EPR spectrum of luminol-TBD-TMPD after addition of H_2_O_2_ (refer to Supplementary Fig. S4) shows no signals, indicating that no ^1^O_2_ is involved in the CL-reaction. As evident from the EPR spectrum of luminol-TBD-DMPO after addition of H_2_O_2_ (depicted in Fig. 4b), ^•^CH_3_, ^•^OH and O_2_^•−^ are generated during the CL-reaction of the luminol-TBD-system. Based on these results, we propose the following reaction mechanism, as illustrated in Fig. 5: In solution, TBD and luminol are present in their de- (protonated) species, the mono-anion-species of luminol and TBDH + (Fig. 5a)^29^. As soon as H_2_O_2_ is added, TBDH^+^ reveals its peroxidase mimetic properties, leading to the decomposition of H_2_O_2_. The TBD is simultaneously regenerated, underpinning the catalytic action of the TBD as coreactant. Since the reaction is carried out in non-anhydrous DMSO, DMSO itself reacts with oxygen and hydroxyl anions to O_2_^•−^ (Fig. 5b). During the DMSO-reaction, the methyl radicals detected in the EPR spectrum are generated, yet without the DMPO they immediately react with oxygen to form the dimethyl peroxide. Finally, the generated ROS oxidize the luminol-mono-anion to the luminol radical anion (LRA). In the presence of ^•^OH and H_2_O_2_, the LRA is further oxidized to the hydroperoxide species via aminodiazaquinone. The direct oxidation of LRA to the hydroperoxide species takes place in the presence of O_2_^•^. Next, the endoperoxide species is formed, nitrogen is released and the excited 3-aminophthalate is formed, which returns to the ground state by the emission of visible light (Fig. 5c)^29^. Spectroscopic (NMR and GC-MS) characterization of the luminol-TBD-system support the proposed mechanism. Directly after the addition of TBD to luminol, a change in the magnetic resonances of the luminol protons d – f in the ^1^H NMR spectrum (refer to Fig. 6) is observed. Since the resonances of protons g from luminol and proton c from TBD are no longer observed and the resonance of proton h shifted to h’, (de-) protonation of TBD and luminol can be assumed. Critically, after the addition of H_2_O_2_, new resonances between 7.3 and 6.7 ppm can be assigned to the oxidized luminol species 3-APA (i – m). The resonance of the NH-proton c of TBD should reappear, however the resonances of the H_2_O_2_-protons overlap exactly with this ppm-range. Nevertheless, the resonances of the TBD-protons a and b reveal no change after addition of luminol and H_2_O_2_, thus the catalytic character of the superbase is emphasized. Furthermore, all resonances in the ^13^C NMR spectrum (Supplementary Fig. S5) can be assigned to luminol, TBD and 3-APA. The GC-MS spectra (refer to Supplementary Figs S6 and S7) underpin the formation of 3-APA. The found mass (163.0 g mol^−1^) from the GC-MS analysis fits to the calculated mass (163.03 g mol^−1^) of the 3-aminophthalic anhydride, which is formed during the ionization process (Supplementary Fig. S6). Although not every signal in the GC measurements could be assigned (green box in Supplementary Fig. S7), clearly no signal of luminol in the oxidized luminol-TBD-system is observed (red box in Supplementary Fig. S7), but a signal of the new product 3-APA (blue box in Supplementary Fig. S7). Remarkably, the two-component system consisting only of luminol and an organic superbase emits photons in such intensity that naked human eye is able to detect the CL triggered by H_2_O_2_. Without any instrument, the emission of light can be observed in a common laboratory under normal working conditions, as shown in the Supplementary video online. The superbase thus combines the necessary basic and catalytic properties in one molecule to enable CL under mild conditions. With mild conditions the CL-performance of a simple two-component-system (luminol and TBD) at ambient temperature in a low-toxic solvent (DMSO) is meant. Since TBD is considered as coreactant, 5.0 eq. of the superbase are required to establish the catalytic and the basic environment for the luminol-CL.

## Conclusion

For the very first time, the organic guanidine-based superbase TBD without any specific buffer solution or additional additive is shown to enable highly efficient CL in the luminol-H_2_O_2_ system as coreactant. The mechanism underpinning TBDs action on the luminol-CL was investigated via chemiluminescence, EPR, 1D/2D NMR, GC-MS and UV/Vis spectroscopy. We submit that TBD promotes as peroxidase mimetic the decomposition of H_2_O_2_ to generate OH^•^ and O_2_^•−^ radicals, which oxidize luminol to the 3-APA under mild conditions, leading to a strong visible CL. Besides possible applications in sensor technology using the luminol-CL, the luminol-TBD-CL with an emission maximum at λ_max_ = 480 nm might be used in photochemistry as a trigger for photoreactions in the visible light range. Critically, with the implementation of superbases to the luminol-reaction, the way is paved for the development of polymeric self-reporting CL-systems. In contrast to conventional bases such as KOH, organic superbases allow the synthesis of monomers for the implementation within multi-functionalized polymers. Thus, the design of a wide range of low-cost materials with interesting properties for self-reporting CL-sensors can be envisaged.

## Experimental Section

### Chemicals

Luminol (Alfa Aesar, 98%), 1,5,7-triazabicyclo[4.4.0]dec-5-ene (TBD, Sigma Aldrich, 98%), 1,1,3,3-tetramethylguanidine (TMG, abcr, 99%), 1,8-diazabizyclo[5.4.0]undec-7-ene (DBU, Sigma Aldrich, ≥99.0%), dimethylsulfoxide (DMSO, Fisher, >99%), hydrogen peroxide (Roth, 35%), sodium azide (NaN_3_, AJA, 99%), thiourea (TU, Scharlau, etra pure), L-ascorbic acid (AA, Sigma Aldrich, 98%, suitable for cell culture), superoxide dismutase (SOD,), 2,2,6,6-tetramethyl-4-piperidone (TMPD, Sigma Aldrich, 95%), 5,5-dimethyl-1-pyrroline N-oxide (DMPO, Sigma Aldrich, ≥97%). All chemicals were used as received.

### EPR spectra

Were recorded on a Magnet Tech MiniScope MS400 spectrometer at ambient temperature in DMSO. The following parameters were applied: B0-field = 336.9748 ± 10.1079 mT, sweep time = 30 s, modulation = 0.01 mT, microwave attenuation = 10.0 db, gain mantisse = 4, gain exponent = 1, number of points = 4096, number of passes = 1.

### NMR spectra

Were recorded on Bruker Avance 400 MHz. Spectra were referenced on residual solvent signal according to Nudelman *et al*.^52^: 2.50 ppm for DMSO-d6. The deuterated solvent was purchased from Euriso-TOP and used without further purification.

For the NMR measurement of luminol-TBD + H_2_O_2_, a stock solution of luminol (*c* = 0.024 mol L^−1^) and TBD (*c* = 0.120 mol L^−1^) in DMSO-d6 was prepared. The CL reaction was triggered with 0.1 mL H_2_O_2_ (*c* = 1 mol L^−1^). After 5 min stirring, 0.6 mL were withdrawn from the solution and put in an NMR tube for analysis.

### UV/Vis spectra

Were recorded on a Cary 100 UV-Visible Spectrometer (Agilent Technologies, USA) possessing a tungsten halogen light source (190 to 900 nm, accuracy +/−2 nm) and a R928 PMT detector. For the measurement, luminol was dissolved in DMSO (*c* = 7.5 × 10^–5^ mol L^−1^), and different equivalents of the superbase were added. The H_2_O_2_ was directly added to the quartz cuvette and the sample was analyzed immediately in the range from 260 to 500 nm. The absorbance of luminol was normalized to 1, all other absorbances were referred to the Luminol-absorbance.

### Chemiluminescence spectra

Were recorded on a Varian Cary Eclipse fluorescence spectrometer in the Bio-/Chemiluminescence mode. The CL emission intensity was recorded in dependence on the wavelength from 350 to 800 nm (scan rate = 600 nm min^−1^, averaging time = 0.1 s, emission slit = 5.0 nm, detector voltage = 800 V) with a luminol concentration of 7.5 × 10^−2^ mol L^−1^. For the kinetic measurements, the emission wavelength was set to 480 nm, according to λ_max_ from the emission spectra. After 15–20 seconds, 0.1 mL H_2_O_2_ (1 mol L^−1^) were directly to the quartz cuvette, mixed and the CL was recorded for 6 minutes. The intensity of the Luminol-TBD-system was normalized to 1, all other intensities were referred to the intensity of the Luminol-TBD-system.

#### Naked-eye detection

For the naked-eye detection of the CL, luminol was dissolved in DMSO with a concentration of 7.5 × 10^−2^ mol L^−1^, then 5.0 eq. of TBD were added. To trigger the CL, 0.1 mL H_2_O_2_ (1 mol L^−1^) were added.

#### GC - MS measurements

A Varian 431 GC instrument with a capillary column FactorFourTM VF-5 ms (30 m·0.25 mm·0.25 μm) and a Varian 210 ion trap mass detector were used. Scans were performed from 40 to 650 m/z at rate of 1.0 scans·s^−1^. The oven temperature program was: initial temperature 95 °C, hold for 1 min, ramp at 15 °C·min^−1^ to 200 °C, hold for 2 min, ramp at 15 °C·min^−1^ to 325 °C, hold for 5 min. Measurements were performed in split–split mode (split ratio 50:1) using helium as the carrier gas (flow rate 1.0 mL·min^−1^).

## Supplementary information

A Guanidine-Based Superbase as Efficient Chemiluminescence Booster

Supplementary Video

## Data Availability

The data that support the findings of this study are available within the article and its Supplementary Information file online, and from the corresponding authors upon reasonable request.

## References

[1] Simoni D, Rondanin R, Morini M, Baruchello R, Invidiata FP (2000). 1,5,7-Triazabicyclo[4.4.0]dec-1-ene (TBD), 7-methyl-TBD (MTBD) and the polymer-supported TBD (P-TBD): three efficient catalysts for the nitroaldol (Henry) reaction and for the addition of dialkyl phosphites to unsaturated systems. Tetrahedron Lett..

[2] Sabet-Sarvestani H, Eshghi H, Izadyar M (2017). A theoretical study on the efficiency and role of guanidines-based organic superbases on carbon dioxide utilization in quinazoline-2,4(1H, 3H)-diones synthesis. Struct. Chem..

[3] Volpe C, Meninno S, Capobianco A, Vigliotta G, Lattanzi A (2019). 1,5,7-Triazabicyclo[4.4.0]dec-5-ene (TBD) Triggered Diastereoselective [3+2] Cycloaddition of Azomethine Imines and Pyrazoleamides. Adv. Synth. Catal..

[4] Porahmad N, Baharfar R (2018). Graphene oxide covalently functionalized with an organic superbase as highly efficient and durable nanocatalyst for green Michael addition reaction. Res. Chem. Intermed..

[5] Ye W, Xu J, Tan C-T, Tan C-H (2005). 1,5,7-Triazabicyclo[4.4.0]dec-5-ene (TBD) catalyzed Michael reactions. Tetrahedron Lett..

[6] Trofimov BA (2017). Efficient switching from the 2,3′- to 2,2′-bipyrrole scaffold via the recyclization of 1-(benzoylmethylanilino)-3-imino-3H-2-cyanopyrrolizines: Crucial effect of the DBU organic superbase. Tetrahedron Lett..

[7] Simoni D (2000). Strong Bicyclic Guanidine Base-Promoted Wittig and Horner−Wadsworth−Emmons Reactions. Org. Lett..

[8] Ruiz-Cantu LA (2019). Synthesis of Methacrylate-Terminated Block Copolymers with Reduced Transesterification by Controlled Ring-Opening Polymerization.

[9] Pratt RC, Lohmeijer BGG, Long DA, Waymouth RM, Hedrick JL (2006). Triazabicyclodecene:  A Simple Bifunctional Organocatalyst for Acyl Transfer and Ring-Opening Polymerization of Cyclic Esters. J. Am. Chem. Soc..

[10] Taylor JE, Bull SD, Williams JMJ (2012). Amidines, isothioureas, and guanidines as nucleophilic catalysts. Chem. Soc. Rev..

[11] Kiesewetter MK (2009). Cyclic Guanidine Organic Catalysts: What Is Magic About Triazabicyclodecene?. J. Org. Chem..

[12] Fu X, Tan C-H (2011). Mechanistic considerations of guanidine-catalyzed reactions. Chem. Commun..

[13] Watanabe N, Wakatsuki A, Ijuin HK, Kabe Y, Matsumoto M (2018). Organic superbase-induced chemiluminescent decomposition of a hydroxyaryl-substituted dioxetane: Unique effect of a bifunctional guanidine base on the chemiluminescence profile of a bicyclic dioxetane bearing a 4-(benzoxazol-2-yl)-3,5-dihydroxyphenyl moiety. Tetrahedron Lett..

[14] Castagnolo D, Schenone S, Botta M (2011). Guanylated Diamines, Triamines, and Polyamines: Chemistry and Biological Properties. Chem. Rev..

[15] Treat NJ (2012). Guanidine-Containing Methacrylamide (Co)polymers via aRAFT: Toward a Cell-Penetrating Peptide Mimic. ACS Macro Lett..

[16] Funhoff AM (2004). Poly(3-guanidinopropyl methacrylate):  A Novel Cationic Polymer for Gene Delivery. Bioconjugate Chem..

[17] Szterk A, Roszko M, Górnicka E (2013). Chemical Stability of the Lipid Phase in Concentrated Beverage Emulsions Colored with Natural β-Carotene. J. Am. Oil Chem. Soc..

[18] Al Lawati HAJ (2013). Flow-based analysis using microfluidics–chemiluminescence systems. Luminescence.

[19] Szterk A, Roszko M, Sosińska E, Derewiaka D, Lewicki PP (2010). Chemical Composition and Oxidative Stability of Selected Plant Oils. J. Am. Oil Chem. Soc..

[20] Szterk A, Lewicki PP (2010). A New Chemiluminescence Method for Detecting Lipid Peroxides in Vegetable Oils. J. Am. Oil Chem. Soc..

[21] Dodeigne C, Thunus L, Lejeune R (2000). Chemiluminescence as diagnostic tool. A review. Talanta.

[22] Luo M (2012). Chemiluminescence biosensors for DNA detection using graphene oxide and a horseradish peroxidase-mimicking DNAzyme. Chem. Commun..

[23] Khan P (2014). Luminol-Based Chemiluminescent Signals: Clinical and Non-clinical Application and Future Uses. Appl. Biochem. Biotechnol..

[24] Karabchevsky A, Mosayyebi A, Kavokin AV (2016). Tuning the chemiluminescence of a luminol flow using plasmonic nanoparticles. Light: Science &Amp; Applications.

[25] Ramsthaler F, Schlote J, Gehl A, Cappel-Hoffmann S, Kettner M (2018). Detectability, visualization, and DNA analysis of bloodstains after repainting the walls. Int. J. Legal med..

[26] Li H, Liu C, Wang D, Zhang C (2017). Chemiluminescence cloth-based glucose test sensors (CCGTSs): A new class of chemiluminescence glucose sensors. Biosens. Bioelectron..

[27] Zhang W (2019). Chemiluminescence chitosan hydrogels based on the luminol analog L-012 for highly sensitive detection of ROS. Talanta.

[28] Roswell, D. F. & White, E. H. In *Methods Enzymol*. Vol. 57, 409–423 (Academic Press, 1978).

[29] Roda, A. *Chemiluminescence and Bioluminescence: Past, Present and Future*. (Royal Society of Chemistry, 2011).

[30] Qin W, Zhang ZJ, Wang FC (1998). Chemiluminescence flow system for the determination of Fe(II) and Fe(III) in water. Fresenius J. Anal. Chem..

[31] Chen L (2012). A novel chemiluminescence immunoassay of staphylococcal enterotoxin B using HRP-functionalised mesoporous silica nanoparticle as label. Food Chem..

[32] Giokas DL, Vlessidis AG, Tsogas GZ, Evmiridis NP (2010). Nanoparticle-assisted chemiluminescence and its applications in analytical chemistry. TrAC, Trends Anal. Chem..

[33] Choi HN, Han JH, Park JA, Lee JM, Lee W-Y (2007). Amperometric Glucose Biosensor Based on Glucose Oxidase Encapsulated in Carbon Nanotube–Titania–Nafion Composite Film on Platinized Glassy Carbon Electrode. Electroanalysis.

[34] Tsaplev YB (2012). Chemiluminescence determination of hydrogen peroxide. J. Anal. Chem..

[35] Marques MPC, de Carvalho CCCR, Cabral JMS, Fernandes P (2010). Scaling-up of complex whole-cell bioconversions in conventional and non-conventional media. Biotechnol. Bioeng..

[36] Ramesh H (2016). Measurement of oxygen transfer from air into organic solvents.

[37] Wang S (2016). Enzyme Stability and Activity in Non-Aqueous Reaction Systems: A Mini Review. Catalysts.

[38] Wildes PD, White EH (1973). Differences between excited states produced chemically and photochemically. Ion pairs of excited states derived from luminol. J. Am. Chem. Soc..

[39] White EH, Zafiriou O, Kagi HH, Hill JHM (1964). Chemilunimescence of Luminol: The Chemcial Reaction. J. Am. Chem. Soc..

[40] White EH, Bursey MM (1964). Chemiluminescence of LUminol and Related Hydrazides: The Light Emission Step. J. Am. Chem. Soc..

[41] Shi M-J, Cui H (2006). Electrochemiluminescence of luminol in dimethyl sulfoxide at a polycrystalline gold electrode. Electrochim. Acta.

[42] Watanabe N, Wakatsuki A, Ijuin HK, Kabe Y, Matsumoto M (2018). Organic superbase-induced chemiluminescent decomposition of a hydroxyaryl-substituted dioxetane: Unique effect of a bifunctional guanidine base on the chemiluminescence profile of a bicyclic dioxetane bearing a 4-(benzoxazol-2-yl)-3,5-dihydroxyphenyl moiety. Tetrahedron Lett..

[43] Vasilescu M, Constantinescu T, Voicescu M, Lemmetyinen H, Vuorimaa E (2003). Spectrophotometric Study of Luminol in Dimethyl Sulfoxide–Potassium Hydroxide. J. Fluoresc..

[44] Kwon MS (2014). Design principles of chemiluminescence (CL) chemodosimeter for self-signaling detection: luminol protective approach. RSC Advances.

[45] Johnson, I. *The Molecular Probes Handbook: A Guide to Fluorescent Probes and Labeling Technologies, 11th Edition*. (Life Technologies Corporation, 2010).

[46] Halliwell B (1991). Reactive oxygen species in living systems: Source, biochemistry, and role in human disease. Am. J. Med..

[47] Merenyi G, Lind JS (1980). Role of a peroxide intermediate in the chemiluminescence of luminol. A mechanistic study. J. Am. Chem. Soc..

[48] He L (2018). *In Situ* Synthesis of Gold Nanoparticles/Metal–Organic Gels Hybrids with Excellent Peroxidase-Like Activity for Sensitive Chemiluminescence Detection of Organophosphorus Pesticides. ACS Applied Materials & Interfaces.

[49] Sun M (2017). Radical-Mediated Spin-Transfer on Gold Nanoclusters Driven an Unexpected Luminescence for Protein Discrimination. Anal. Chem..

[50] Bancirova M (2011). Sodium azide as a specific quencher of singlet oxygen during chemiluminescent detection by luminol and Cypridina luciferin analogues..

[51] Prasad AK, Mishra PC (2017). Scavenging of superoxide radical anion and hydroxyl radical by urea, thiourea, selenourea and their derivatives without any catalyst: A theoretical study. Chem. Phys. Lett..

[52] Fulmer GR (2010). NMR Chemical Shifts of Trace Impurities: Common Laboratory Solvents, Organics, and Gases in Deuterated Solvents Relevant to the Organometallic Chemist. Organometallics.

