# Supplementary material for: A Guanidine-Based Superbase as Efficient Chemiluminescence Booster
Source: Sci Rep. 2019 Oct 10;9:14519. doi: 10.1038/s41598-019-51105-z (PMC6786995; doi:10.1038/s41598-019-51105-z)
Supplement: Supplementary file 1 — A Guanidine-Based Superbase as Efficient Chemiluminescence Booster [file 41598_2019_51105_MOESM1_ESM.pdf]

A Guanidine-Based Superbase as Efficient Chemiluminescence Booster

Supplementary Information

Christina M. Geiselhart,[a,b] Christian W. Schmitt,[c] Philipp Jöckle,[d] Hatice Mutlu*[a,b] and Christopher Barner-Kowollik*[b,c]

[a]Soft Matter Synthesis Laboratory, Institut für Biologische Grenzflächen, Karlsruhe Institute of Technology (KIT), Hermann-von-Helmholtz-Platz 1, 76344, Eggenstein-Leopoldshafen, Germany.

[b]Macromolecular Architectures, Institut für Technische Chemie und Polymerchemie, Karlsruhe Institute of Technology (KIT), Engesserstraße 18, 76128, Karlsruhe, Germany.

[c]School of Chemistry, Physics and Mechanical Engineering and Institute of the Future Environments, Queensland University of Technology (QUT), 2 George Street, QLD 4000, Brisbane, Australia.

[d]Molecular Physical Chemistry, Institute of Physical Chemistry, Karlsruhe Institute of Technology (KIT), Fritz-Haber-Weg 2, 76131 Karlsruhe, Germany

A Content

A Content ... 2
B Additional Information – Non-substituted vs substituted Guanidines ... 2
C Additional Data and Figures ... 3
C.1. CL emission ... 3
C.2. UV-Vis spectra ... 4
C.3. EPR spectrum of Luminol-TBD-TMPD-H_2O_2 ... 4
C.4. NMR spectra ... 5
C.5. GC – Chromatograms ... 5
D References ... 6

B Additional Information – Non-substituted vs substituted Guanidines

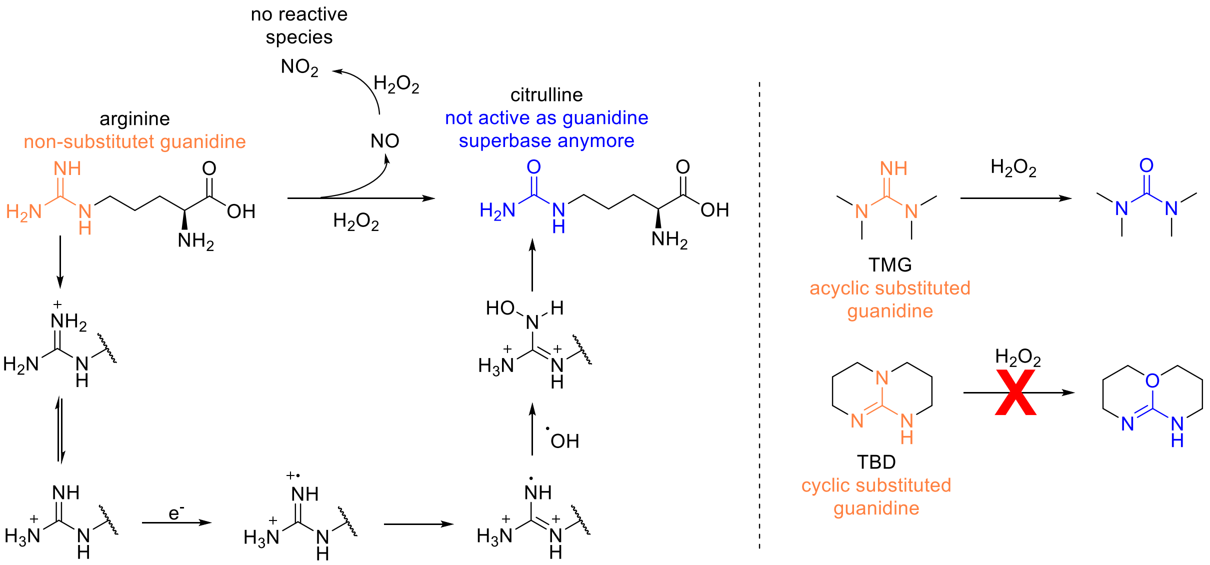

Figure S1: Oxidation mechanism of arginine, a non-substituted guanidino compound, in contrast to acyclic / cyclic substituted guanidino compounds (TMG / TBD).

In the present work, organic superbases, namely TMG, TBD and DBU, were implemented to the chemiluminescence (CL) reaction of luminol. As explained in the main text, the superbases are supposed to function both as base and catalyst. Thus, it is important they keep their properties during the oxidation process for the CL reaction in the presence of H_2O_2. However, it has been reported that free guanidine functionalities are converted to urea moieties in the presence of oxidants,[1,2] which are not active as guanidine superbases anymore. The proposed mechanism is displayed in Figure S1 (left side), exemplary shown for arginine, a natural amino acid which incorporates a non-substituted guanidine moiety. In the presence of an oxidant such as H_2O_2, the activated guanidine moiety reacts with a hydroxyl radical and the urea-containing citrulline is formed. The citrulline no longer functions as base and catalyst, thus being not suitable for the CL reaction of luminol. Furthermore, the NO-

species generated during the oxidation process consumes the H_2O_2 to form the non-reactive NO_2, preventing any CL when implemented to the CL reaction of luminol. Therefore, substituted guanidines (TMG and TBD) were chosen for the implementation to the CL reaction process of luminol. The substituents prevent the charge delocalization and thus, the conversion of the substituted guanidine superbases to the urea compounds, as illustrated in Figure S1 (right side). Critically, the structure of the superbases play an important role in the context of the luminol-Cl, since the substituted guanidine superbases used in the current work could successfully be implemented to the CL reaction of luminol, as proven by the results discussed in the main text.

C Additional Data and Figures

C.1. CL emission

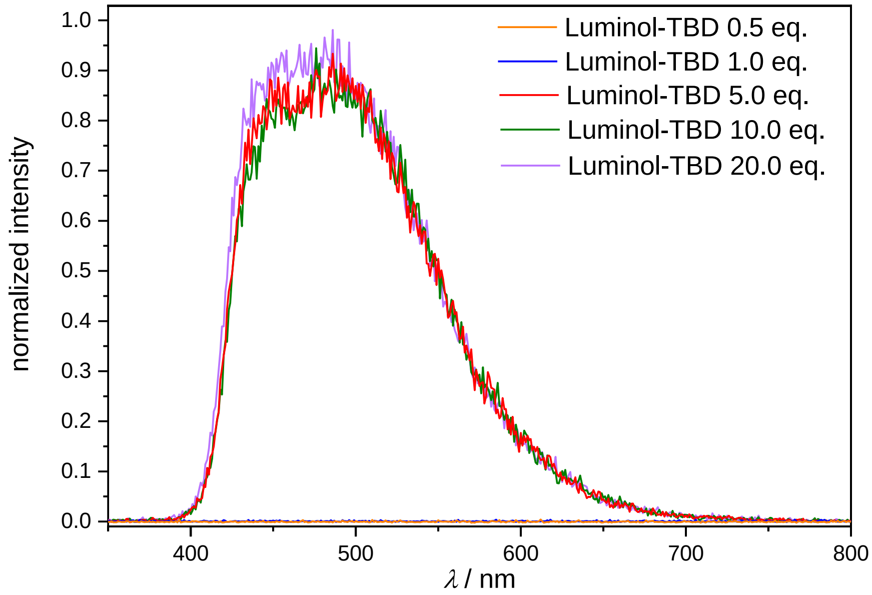

Figure S2: CL emission of luminol (c=7.5*10^{-2} mol L^{-1}) in DMSO with different concentrations of TBD (c(0.5 eq.)=3.75x10^{-2} mol L^{-1}, c(1.0 eq.)=7.5x10^{-2} mol L^{-1}, c(5.0 eq.)=37.5x10^{-2} mol L^{-1}, c(10.0 eq.)=75x10^{-2} m ol L^{-1}, c(20.0 eq.)=150x10^{-2} mol L^{-1}) at ambient temperature, triggered by H_2O_2.

C.2. UV-Vis spectra

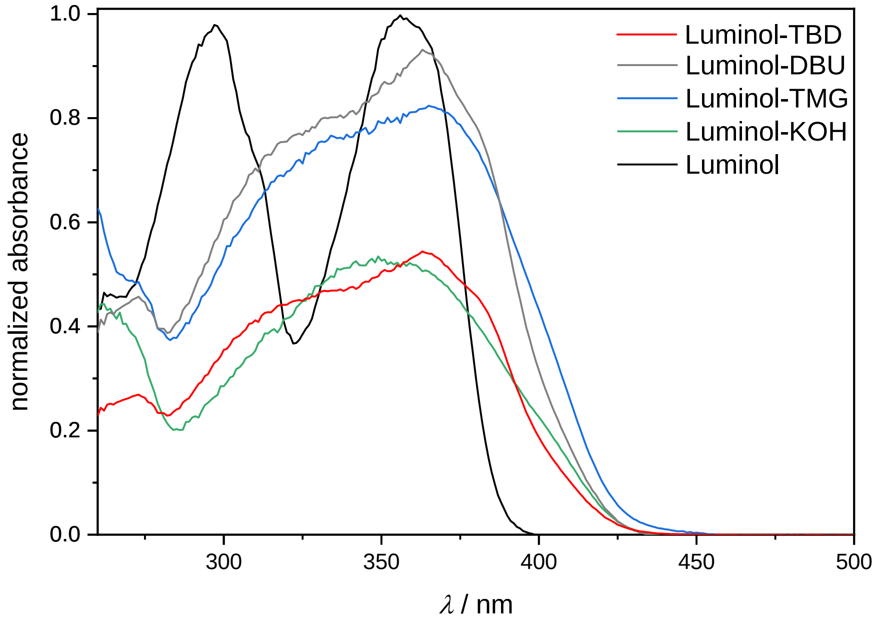

Figure S3: UV/Vis spectra of luminol-CL-systems in DMSO (c(lum)=7.5x10^{-5} mol L^{-1}, c(base)=37.5x10^{-5} mol L^{-1}) at ambient temperature (without the addition of H_2O_2).

C.3. EPR spectrum of Luminol-TBD-TMPD-H_2O_2

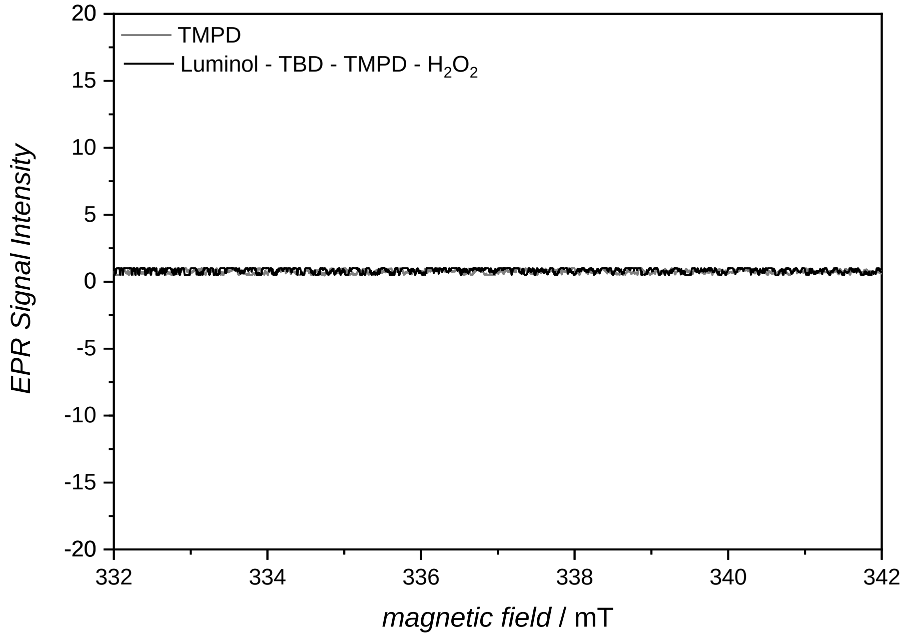

Figure S4: EPR spectra of TMPD (grey line) and the luminol-TBD-system in the presence of TMPD after addition of H_2O_2.

C.4. NMR spectra

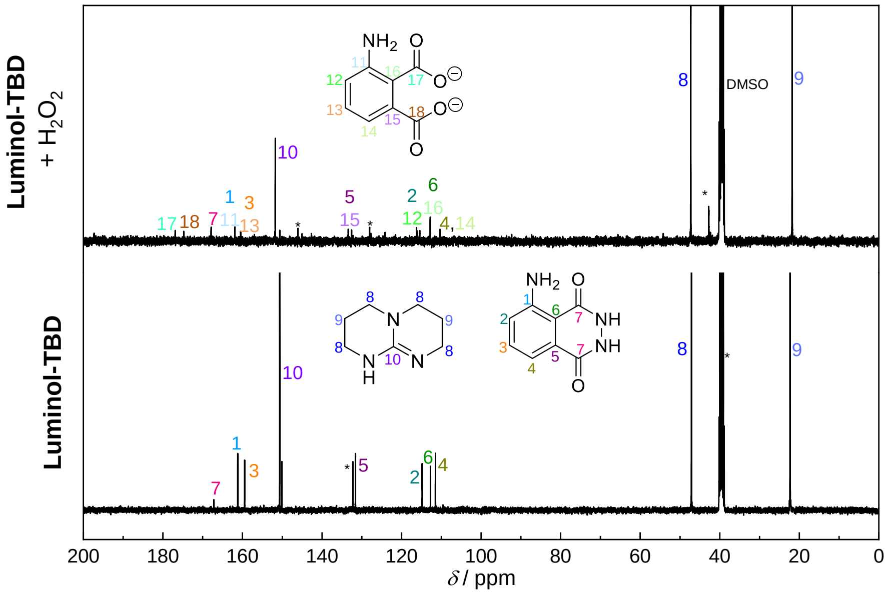

Figure S5: ^{13}C NMR (400 MHz) spectra of Luminol-TBD and Luminol-TBD + H_2O_2 in DMSO-d6.

C.5. GC – Chromatograms

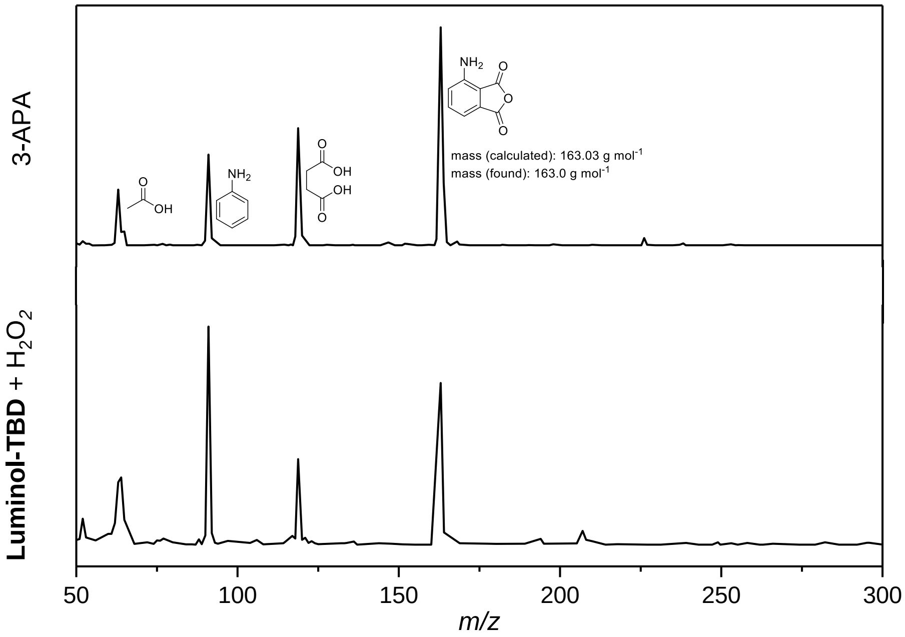

Figure S6: MS spectra of 3-APA and **Luminol-TBD**+H_2O_2 in MeOH.

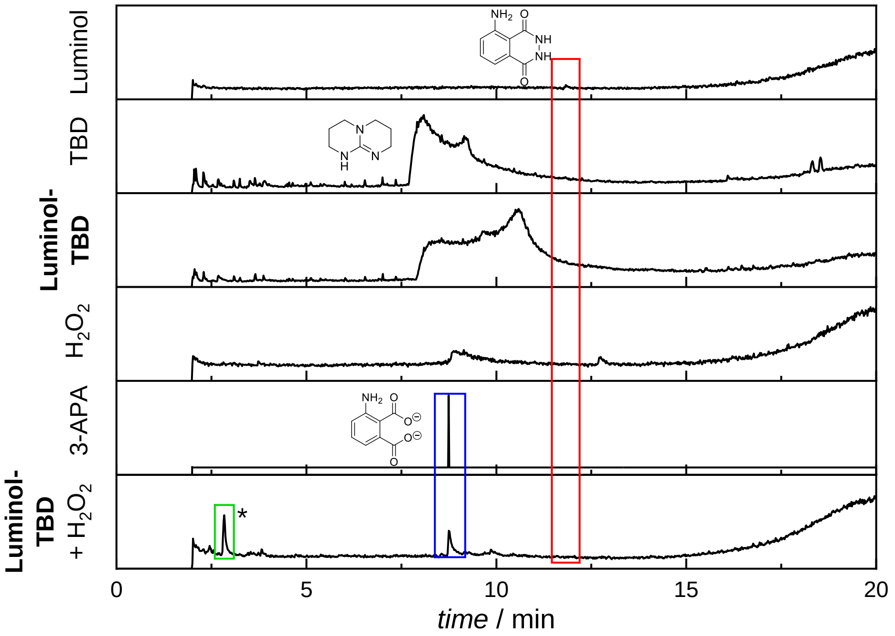

Figure S7: GC – MS - measurements of luminol, TBD, Luminol-TBD, H_2O_2 and Luminol-TBD + H_2O_2 in MeOH.

D References

1 Giroud, C. *et al.* Role of arginine guanidinium moiety in nitric oxide synthase mechanism of oxygen activation. *J. Biol. Chem.* **285**, 7233 (2009).

2 Mori, A., Kohno, M., Masumizu, T., Noda, Y. & Packer, L. Guanidino compounds generate reactive oxygen species. *IUBMB Life* **40**, 135-143 (1996).
